# Global Transcriptome Profiles of Italian Mediterranean *Buffalo* Embryos with Normal and Retarded Growth

**DOI:** 10.1371/journal.pone.0090027

**Published:** 2014-02-28

**Authors:** Maria Strazzullo, Bianca Gasparrini, Gianluca Neglia, Maria Luisa Balestrieri, Romina Francioso, Cristina Rossetti, Giovanni Nassa, Maria Rosaria De Filippo, Alessandro Weisz, Serena Di Francesco, Domenico Vecchio, Maurizio D'Esposito, Michael John D'Occhio, Luigi Zicarelli, Giuseppe Campanile

**Affiliations:** 1 Institute for Animal Production System in Mediterranean Environment, National Research Council, Naples, Italy; 2 Department of Veterinary Medicine and Animal Production, Federico II University, Naples, Italy; 3 Department of Biochemistry, Biophysics and General Pathology, Second University of Naples, Naples, Italy; 4 Institute of Genetics and Biophysics ABT, National Research Council, Naples, Italy; 5 Istituto di Ricovero e Cura a Carattere Scientifico (IRCSS) Neuromed, Pozzilli, Italy; 6 Laboratory of Molecular Medicine and Genomics, Department of Medicine and Surgery, University of Salerno, Baronissi (SA), Italy; 7 Fondazione Istituto di Ricovero e Cura a Carattere Scientifico SDN, Napoli, Italy; 8 Faculty of Agriculture and Environment, The University of Sydney, Camden, NSW, Australia; National Cancer Institute, United States of America

## Abstract

The transcriptome profiles were compared for buffalo embryos with normal growth and embryos with retarded growth on Day 25 after mating. Embryos with retarded growth on Day 25 after mating have a reduced likelihood of undergoing attachment to the uterine endometrium and establishing a pregnancy. Italian Mediterranean buffaloes were mated by AI and on Day 25 underwent trans-rectal ultrasonography to ascertain embryo development. Embryos with an embryonic width (EW)>2.7 mm were classed as normal embryos and embryos with an EW<2.7 mm were classed as retarded embryos. Three buffaloes with embryos of the largest EW (3.7, 3.7 and 3.9 mm) and three buffaloes with embryos of the smallest EW (1.5, 1.6 and 1.9 mm) were slaughtered on Day 27 to recover embryos for transcriptome analysis using a bovine custom designed oligo array. A total of 1,047 transcripts were differentially expressed between embryos with normal growth and embryos with retarded growth. Retarded embryos showed 773/1,047 (74%) transcripts that were down-regulated and 274/1,047 (26%) transcripts that were up-regulated relative to normal embryos; *in silico* analyses focused on 680/1,047 (65%) of the differentially expressed transcripts. The most altered transcripts observed in retarded embryos were associated with membrane structure and function and with metabolic and homeostasis maintenance functions. Other notable functions altered in retarded embryos were developmental processes and in particular nervous system differentiation and function. Specific biochemical pathways such as the complement cascade and coagulation were also altered in retarded embryos. It was concluded from the findings that buffalo embryos with retarded growth on Day 25 after mating show altered gene expression compared with normal embryos, and some de-regulated functions are associated with attachment to the uterine endometrium.

## Introduction

The water buffalo (*Bubalus bubalis*) is a short-day breeder and at higher latitudes females show distinct seasonal changes in reproductive function. Optimal fertility occurs during decreasing day length in autumn and fertility to both natural mating and AI declines appreciably during increasing day length from late-winter to early-spring [1], [2]. The latter period is recognized as the transition phase from the breeding to non-breeding season [1], [2]. Notable features of ovarian function during the transition phase are the reduced size of the corpus luteum, decreased vascularization, and relatively low concentrations of circulating progesterone (P4) [3], [4]. The latter was proposed to be a major factor in the increased incidence of embryonic mortality, which is the primary cause of reduced fertility in buffaloes during the transition phase [5]. Progesterone has been shown to have a fundamental role in embryonic development and implantation in buffalo [2] and also cattle [6]–[8].

A notable feature of embryonic mortality in buffaloes is that the highest incidence occurs between Days 25 and 45 after mating [1], [5], [9], [10]. This represents late embryonic mortality and differs from cattle that typically undergo embryonic mortality between Days 8 and 17 after mating [11],[12]. The latter covers the period during which there is maternal recognition of pregnancy. It was proposed that late embryonic mortality in buffaloes results from the failure of embryonic attachment [2], [13] which is a consequence, at least in part, of reduced concentrations of P4 in circulation from around Day 10 after mating [3].

Buffalo embryos destined to undergo late embryonic mortality show retarded growth at Day 25 after mating compared with embryos that maintain pregnancy [14]. Balestrieri et al. [13] recently reported that the chorioamnion of embryos with retarded growth (embryo width<2.7 mm) had a different proteomic profile to that of embryos with normal growth (embryo width>2.7 mm). Differences in proteome profiles were also reported for the caruncles adjacent to the chorioamnions of embryos with retarded and normal growth. Differentially expressed proteins were related to antioxidant protection, protease inhibition and protein folding [13]. These findings supported other evidence that a disruption of conceptus-uterus interaction, as a result of reduced progesterone, is the major cause of late embryonic mortality in buffaloes [1], [2].

Global transcriptome profiling has been used in cattle to gain insight into the molecular features of embryonic development [7], [15], [16] and embryonic attachment to the uterine endometrium [6], [17]–[19]. As noted above, embryonic mortality in cattle is most likely to occur before the window for maternal recognition of pregnancy closes, which is around Days 16–17. Embryo and uterine transcriptome profiling in cattle has therefore mainly been studied up to this period. In a study carried out on Day 16, 46 transcripts were conceptus-specific and 34 transcripts were endometrium-specific [19]. It was proposed that the differentially expressed transcripts were involved in the maternal recognition of pregnancy. In a second study in cattle, changes were observed from Day 21 to Day 28 in the gene expression profile of conceptus trophoblast membranes and it was similarly proposed that the changes in expression were related to embryonic attachment [20].

The decline in fertility in buffaloes during the transition phase to the non-breeding season has a significant impact on the rate of genetic improvement and distribution of germplasm, which reduces the efficiency of production. An improved understanding of the molecular basis for late embryonic mortality in buffaloes would contribute to fundamental comparative reproductive biology and also lead to strategies to enhance fertility during the transition phase. In the present study, global transcriptome profiling was undertaken for the first time in buffalo embryos that showed either normal or retarded growth at Day 25 after mating.

## Materials and Methods

### Embryo collection

Embryos were obtained from multiparous Italian Mediterranean buffaloes (*Bubalus bubalis*) that had undergone synchronization of ovulation and artificial insemination (AI) during the transition phase to the non-breeding season as described by Balestrieri et al. [13]. The “Ethical Animal Care and Use Committee” of the University of Naples Federico II approved the experimental design and animal treatments (Permit Number: 2013/010858).

Buffaloes underwent ultrasonography on day 25 after AI and were divided in two groups according to the embryo width (> or<than 2.7 mm). Three buffaloes that had normal embryos, i.e. with the largest embryo width and three buffaloes that had retarded embryos, i.e. with the smallest embryo width on Day 25 were slaughtered on Day 27 to collect embryos, as previously described [13]. At slaughter (Day 27) the embryos with the largest EW (3.7, 3.7 and 3.9 mm) had a respective EL of 9.8, 7.5 and 9.8 mm and embryos with the smallest EW (1.5, 1.6 and 1.9 mm) had a respective EL of 5.0, 5.5 and 5.0 mm.

### RNA extraction

After removal from the uterus embryos were measured and placed in a test tube containing RNAlater solution. Total RNA was extracted from each embryo according to the Trizol protocol (Invitrogen). The concentration of RNA was determined with a Nanodrop (NanoDrop,) spectrophotometer and RNA quality was assessed with an Agilent 2100 Bioanalyzer (Agilent Technologies).

### Microarray hybridization

A comparative genomic approach was adopted [21] which utilized a heterologous hybridization technique [22], [23]. The EmbryoGENE bovine transcriptome microarray [24] was chosen for genome-wide analysis. This was a custom designed oligo array, constructed from global transcriptome RNA sequencing (RNA-Seq) experiments and enriched for embryo-specific transcripts and 3′UTR variants.

For each sample (embryo), 200 ng of total RNA were synthesized to Cy3-labeled, linearly amplified cRNA using the One-Color LowInput Quick Amp Labeling kit (Agilent Technologies). The concentration and quality of cRNA was assessed using a Nanodrop (NanoDrop) spectrophotometer. For each of the normal and retarded embryos, three assay replicates were produced and 1.65 µg of Cy3-labeled linearly amplified cRNA was fragmented and hybridized for 17 h to the Agilent bovine (*Bos taurus*) 4×44 k oligonucleotide microarray according to the protocol provided by the manufacturer (Agilent Technologies).

### Array scanning and data analysis

Agilent Feature Extraction (AFE) 11.0.1.1 image analysis software was used to extract intensity from scanned images (file.tiff) obtained after microarray slides reading. The intensity files were loaded into GeneSpring software version 11.5 (Agilent Technologies) for quality control and gene expression analysis. First, the percentile shift normalizing algorithm, shifting to 75 percentile, was applied on the dataset to correct systematic errors. For differential expression analysis, assay replicates and embryo replicates were grouped together, and only genes detected in 100% of assay replicates for each embryo, were considered. The Unpaired t-Test with FDR correction was used to calculate a P-value for each differentially expressed gene, whilst fold-change was used to calculate differential expression between the two conditions. Only genes with adjusted P values≤0.05, and fold-change≥1.5 and ≤-1.5, were regarded as differentially expressed. Microarray data are available in the ArrayExpress database (www.ebi.ac.uk/arrayexpress) under accession number E-MTAB-2064.

### Quantitative RT-PCR analysis

The transcripts to analyze were chosen according to three main criteria: fold change degree, biochemical and functional roles, the availability of working primers, as the RT-PCR primers were designed according to bovine sequences in GenBank. The six up-regulated transcripts analyzed were: annexin A3 (*ANXA3*), serpin peptidase inhibitor, clade E (nexin, plasminogen activator inhibitor type 1), member 1 (*SERPINE1*), keratin 18 (*KRT18*), syntaxin 7 (*STX7*), tetraspanin 8 (*TSPAN8*) and ectonucleotide pyrophosphatase/phosphodiesterase 3 (*ENPP3*). The four down-regulated transcripts were: the complement component C5 (*C5*), D-aspartate oxidase (*DDO*), dihydropyrimidinase (*DPYS*) and myosin X (*MYO10*). Specific primers were designed through the Primer-Blast algorithm (http://www.ncbi.nlm.nih.gov/tools/primer-blast) referring to the corresponding bovine gene sequences available in GeneBank (www.ncbi.nlm.nih.gov/genbank) and through the Blast-primer algorithm.

The primers used for amplification of GAPDH, the reference gene, were designed according to the *Bubalus*-specific sequence. Primer sequences and melting temperature (Tm), amplicons length, and the accession numbers for the bovine and buffalo sequences are shown in Table 1. Each RT-PCR was performed in triplicate using the CFX96 Real-Time PCR (Bio-Rad Laboratories). The reactions were performed in 20 µl final volume with the 2× Master mix Bio-Rad iQ SYBR Green Supermix, 125 µM of each primer and 1 µl of a 1/10 dilution of cDNA obtained through reverse transcription starting from 1 µg of total RNA, DNA free, in 20 µl of reaction volume. The RT-PCR protocol includes 35 amplification cycles. The expression level of each transcript was normalized to GAPDH transcript levels. The relative expression levels in the analyzed tissues were obtained using the 2^−ΔΔ Ct^ method [25].

**Table 1:** Accession number (a), Gene Symbol (b), primers sequence (c), amplicon length (d) and Tm (e) of transcripts validated in qPCR experiments.

a	b	c	d	e
NM_001035325	*ANXA3*	F 5′ GACCCCACCGGCAGTGTTCG 3′ R 5′ GGCATGGCCGATCTCCTGCAT 3′	130 bp	64°
NM_174137	*SERPINE1*	F 5′ GTTTAGGCCGAGCCAGGCGG 3′ R 5′ TGGGATTGTGCCGCACCACG 3′	206 bp	64°
NM_001079629	*TSPAN8*	F 5′ CCTGGGATGCTGTGGTGCCAT 3′ R 5′ ACGATACCTGCCGCCACCTGC 3′	102 bp	64°
NM_001192095	*KRT18*	F 5′ GCAGACCGCTGAGATAGGAG 3′ R 5′ CAAGCTGGCCTTCAGATTTC 3′	109 bp	60°
NM_001077864	*STX7*	F 5′ ACAGAGGACGACCTGCGCCTT 3′ R 5′ TGCGCTGATAGTCTGCCGCC 3′	217 bp	64°
NM_173908	*DDO*	F5′TGTGAAGGCCCTGCCTACCTCC 3′ R5′ CGGGTGAAGCTCCCACAGGTC 3′	102 bp	64°
NM_001101065	*MIOX*	F 5′ CTACACGTCTGGCCCGCTCCT 3′ R 5′ CCCCGAACTGGGCATGCTTCC 3′	104 bp	64°
NM_001166616	*C5*	F 5′CGCAAACGCAGATGACACCCG 3′ R 5′ TGGGGCCTGCCTGAATCCGA 3′	200 bp	62°
NM_001075923	*ENPP3*	F 5′ TGTTGGTGGCTGTGTCACTT 3′ R 5′GGCAGCCCTCTAGTCCTCTA 3′	117 bp	60°
NM_001192214	*DPYS*	F 5′ CCTTCAACGCCTGACTTCCT 3′ R 5′ AGAGCTTTCTGGCAGCTGTT 3′	98 bp	58°
GU324291	*GAPDH*	F 5′ TCACTGGCATGGCCTTCCGC3′ R 5′ GCCCTCTGACGCCTGCTTCAC 3′	122 bp	60°

### Bioinformatic tools and databases

The Database for Annotation, Visualization and Integrated Discovery (DAVID, http://david.abcc.ncifcrf.gov) [26] was used to ascertain major biochemical and functional pathways. The full list of differentially expressed transcripts and the list of the up- and down-regulated genes, respectively, were separately analyzed and compared. The molecular features taken into account for analysis were selected according to a statistical cut off P-value of 0.05, after the Benjamini method correction, and the percentage of genes included in each biological group.

The visualization of the main gene networks involved was obtained through Ingenuity Pathways Analysis (IPA, Ingenuity Systems, http://www.ingenuity.com).

## Results

### Gene expression profiling

**Figure 1:**
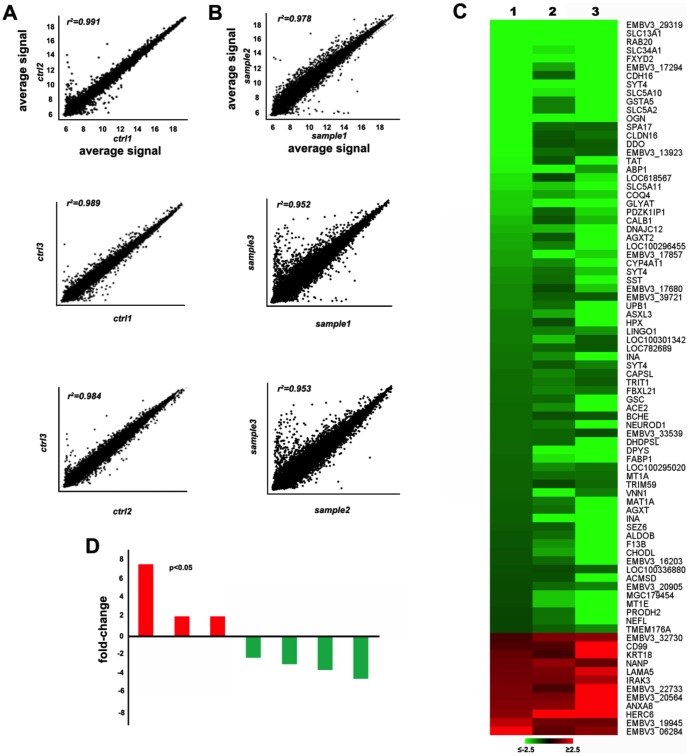
Microarray hybridization experiments. Panel A and panel B show the correlation of the results among the three samples of each group (respectively ctrl  =  normal embryos and sample  =  retarded embryos); Panel C depicts the heat map corresponding to some differential expressed transcripts. The number 1, 2, 3 refer to retarded embryo samples; Panel D shows the relative fold change of some differential expressed transcripts.

The heterologous hybridization of the bovine array showed that, a total of 1,047 transcripts were differentially expressed between embryos with normal growth and embryos with retarded growth (Figure 1). In panel A and B the correlation of the results among the samples of each group (respectively normal and retarded embryo) is shown. Retarded embryos showed 773/1,047 (74%) transcripts that were down-regulated and 274/1,047 (26%) transcripts that were up-regulated relative to normal embryos. A heat map of representative differentially expressed transcripts and a graphic representation are shown in panels C and D of Figure 1.

### Quantitative RT-PCR

**Figure 2:**
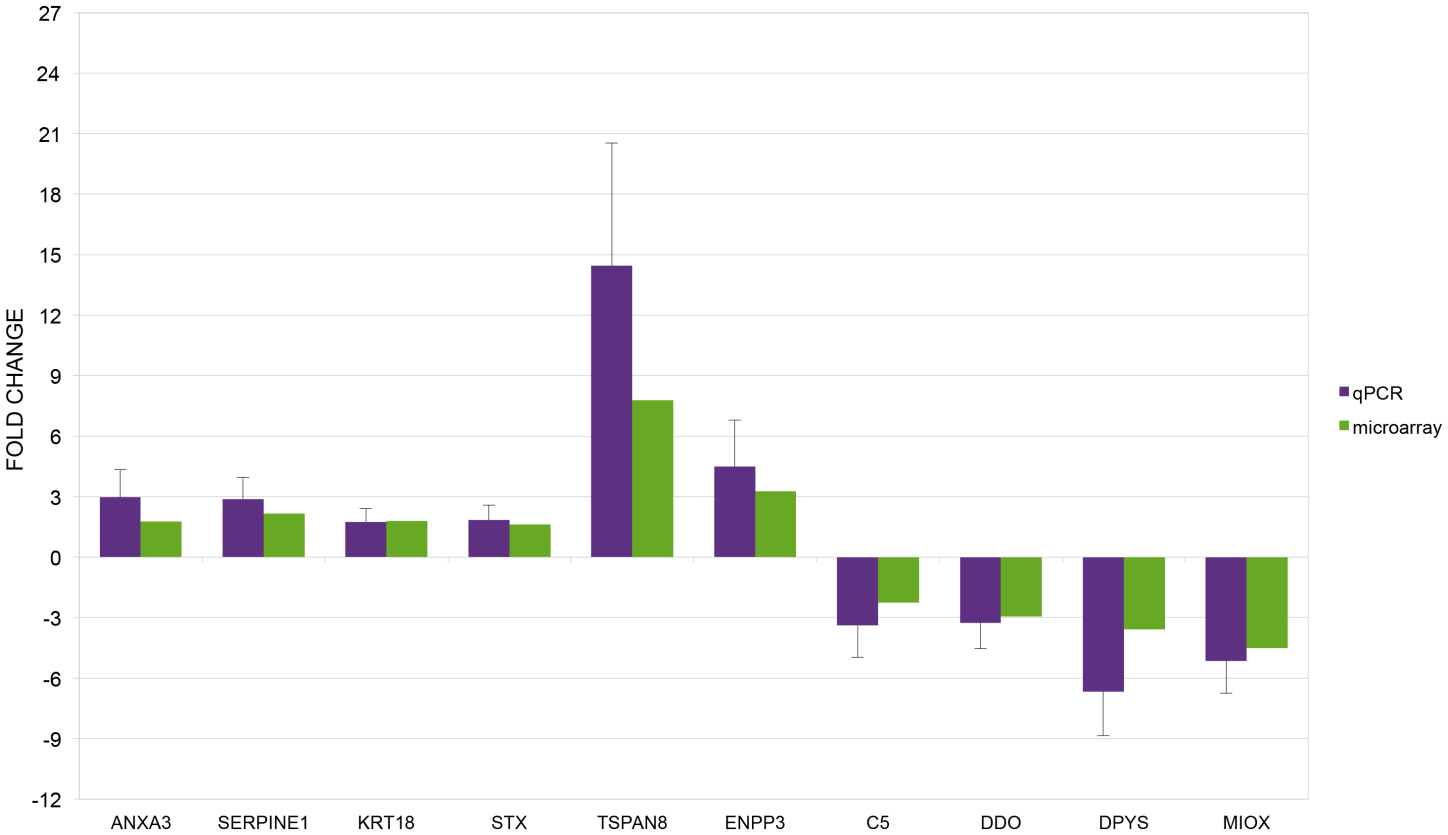
qPCR microarray data validation. Graph of qPCR data for selected genes compared to corresponding microarray data. The results are expressed as the mean + standard deviation of the relative expression of each transcript.

The microarray hybridization results were corroborated by the quantitative RT-PCR analysis of ten transcripts. In Figure 2 are shown the results of qPCR (retarded embryos versus normal embryos) compared to the corresponding results in microarray experiment. There was agreement for all compared transcripts. The direction of the expression change (up- or down-regulation) was confirmed for all transcripts.

### In silico analysis of differentially expressed transcripts

The *in silico* analyses focused on about 680/1,047 (65%) of the differentially expressed transcripts. Of the transcripts not included, about 15% were described as “Novel Transcribed: embryo expressed sequence tags (ESTs)” that at present lack a GenBank annotation; about 10% were only predicted in the bovine genome and lack any functional annotations; and about 7% were alternate forms of the same locus or polymorphic alleles. The Database for Annotation, Visualization and Integrated Discovery (DAVID), and the Ingenuity Pathway Analysis (IPA) tools were used to explore and enlighten the main molecular features and the biochemical networks that were altered in retarded versus normal embryos.

### Database for Annotation, Visualization and Integrated Discovery (DAVID) analysis

**Figure 3:**
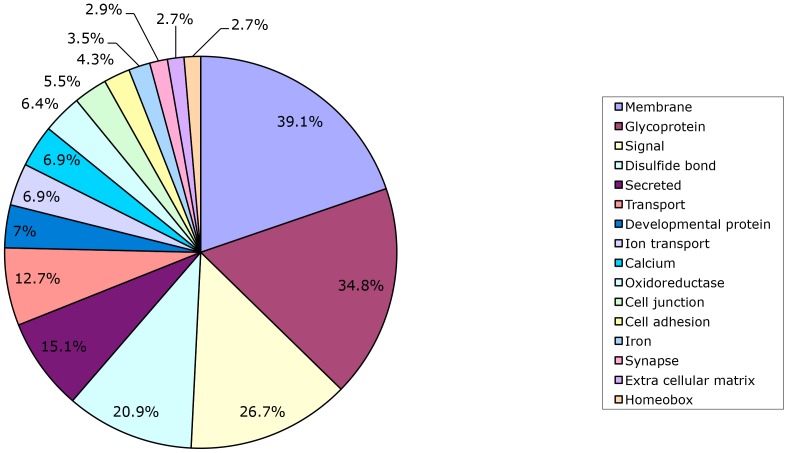
Bioinformatic data analysis. The results of the SP/PIR TOOL analysis of the data are summarized and the class and percentage of transcripts are reported.

A scheme of the main classes of putative transcriptionally altered proteins in retarded and normal embryos is depicted in Figure 3. The more abundant class (39.1%) contains proteins involved in membrane structure and function. A further dissection of this major class showed that a large number of the proteins are glycoproteins, which are also the second most abundant class (34.8%) (Figure 3). The major functions summarized (signaling, molecule secretion, transport of macromolecules and ions, cell-cell junctions, cell adhesion, synapses) are associated with both basic and specialized membrane functions. The group of developmental (7.0%) and homeobox (2.7%) proteins are involved in the regulation and coordination of embryonic growth. All the features and functional classes described in Figure 3 were further interrogated and clarified from the analysis of microarray data through the Gene Ontology Tool and the results are shown in Table 2. The most abundant classes related to developmental processes and in particular cell and tissue differentiation, including cellular components and extracellular matrix. Over 80 de-regulated transcripts in retarded embryos were related to nervous system development. This included neuron cell differentiation and other functional features such as ion transport, cell-cell signaling and response to external stimuli (hormones and growth factors). The latter mechanisms are also more generally associated with the maintenance of cellular homeostasis, which would seem to have been compromised in the retarded embryos (Table 2). Pivotal aspects of basal metabolism such as oxidation-reduction reactions, biosynthetic processes, and amino acid and lipid metabolism, also differed between normal and retarded embryos (Table 2). Transcription factors and regulatory proteins also seemed to differ between normal and retarded embryos (Table 2).

**Table 2:** Gene Ontology analysis for molecular functions, subclasses, and percentage, of the differentially expressed genes (DEGs), for normal and retarded buffalo embryos on Day 27 of development.

Molecular function	Subclasses	DEGs (%)
Developmental process	Ion/cation transport; Neuron differentiation; Cell adhesion; Oxidation reduction	25.0
Homeostatic process	Neurological processes; Ion homeostasis; Regulation of hormone levels; Cell-cell signalling	16.3
Cell differentiation	Cellular process; Cellular component biogenesis; Biological regulation	15.0
Nervous system development	Cell differentiation; Neurogenesis	12.7
Morphogenesis	Multicellular organismal development; Cellular component morphogenesis	11.9
Response to external stimuli	Response to stress; Response to chemical stimuli; Response to wounding; Inflammatory response; Blood coagulation	10.1
Organic acid metabolic process	Biosynthetic process; Amino acid metabolic process; Lipid metabolic process	7.5

### Ingenuity Pathway Analysis (IPA)

**Figure 4:**
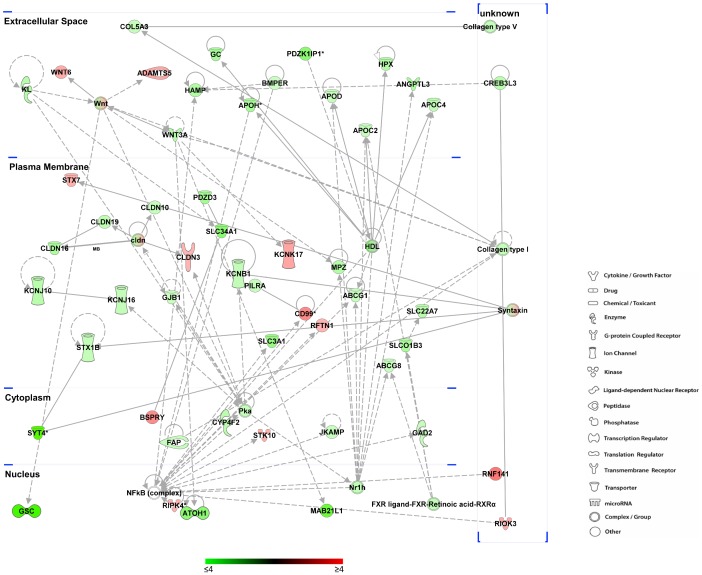
Lipid metabolism network. The scheme shows the altered transcripts involved in lipid metabolism pathways (according to IPA algorithm). The putative cellular and extracellular localization of each transcript is also shown. The colour of each transcript indicates the expression status: the colour scale from green to red indicates the degree of down- and up-regulation respectively. The legend of shapes correspondence is also reported.

The Ingenuity Pathway Analysis provided further detail on the functional pathways expressed between normal and retarded embryos, visualizing the molecular networking among different pathways and cellular compartments. The network of altered transcripts involved in lipid metabolism (Figure 4) is a clear example of the large range of functions altered in retarded embryos. The transcripts (57) described in this network are associated with all cellular compartments and a large number of the proteins are localized in the plasma membrane. These proteins are involved in a wide range of pathways and functions including ion, vitamin and protein transport (KCNB1, GC, SLC34A1), tight junctions (*CLDN10*), transcriptional regulators (GSC,), enzymes (GAD2) and apoliproteins (APOD, APOH).

Fifteen of the differentially expressed transcripts in retarded embryos (12 down-regulated and 3 up-regulated) are associated with the complement/coagulation cascade (Table 3). These included members of the serine protease family (*SERPINE1, SERPIND1 and SERPINA1*). SERPINE1 interacts with PLAT to regulate the activity of plasminogen (PLG) that in turn regulates the complement cascade (C5, C8A) (Table 3). The transcripts of *SERPINE1* and *C5* are two of the ten transcripts whose altered expression was confirmed by qRT-PCR in both normal and retarded embryos (Figure 2, Table 1).

**Table 3:** Coagulation/complement cascade genes.

Gene Symbol	Description	Direction of change
*CD55*	CD55 molecule, decay accelerating factor for complement	+2.04
*CPB2*	carboxypeptidase B2 (plasma)	−3.25
*F2*	coagulation factor II (thrombin)	−1.88
*F9*	coagulation factor IX	−3.9
*F13B*	coagulation factor XIII, B polypeptide	−4.86
*C5*	complement component 5	−2.24
*C8A*	complement component 8, alpha polypeptide	−1.70
*C8B*	complement component 8, beta polypeptide	−1.72
*FGB*	fibrinogen beta chain	−1.59
*MBL2*	mannose-binding lectin (protein C) 2, soluble	−1.54
*PLG*	plasminogen	−1.89
*PLAU*	plasminogen activator, urokinase	+1.76
*SERPINA 5*	SERPINE peptidase inhibitor, clade A (antitrypsin) 5	−1.75
*SERPIND1*	SERPIN peptidase inhibitor, clade D (heparin cofactor) 1	−1.97
*SERPINE1*	SERPIN peptidase inhibitor, clade E (nexin, plasminogen activator inhibitor type 1) member 1	+2.16

Gene identifier, description, and direction of change, of the differentially expressed genes involved in the coagulation/complement cascade for retarded compared with normal buffalo embryos on Day 27 of development are reported.

## Discussion

Buffalo embryos that undergo late embryonic mortality have retarded growth on Day 25 of development compared with embryos that continue to develop [13], [14]. A comparison was therefore made of the transcriptome profiles for normal and retarded embryos on Day 27. At this stage of development embryos are preparing to attach to the uterine endometrium which represents a critical phase in the establishment of a pregnancy. Analysis of the transcriptome revealed that retarded embryos undergo extensive changes in the expression of genes involved in a wide range of biological mechanisms associated with developmental processes and cell differentiation. The large number of abnormally expressed protein associated to membrane structure and functions, correlates with the abundance and heterogeneity of this class of proteins [27].

The altered transcripts are involved in signalling, ion transport and molecule secretion, cell junction and adhesion, extra cellular matrix components. All these functions allow the response to internal and external stimuli, contributing to the maintenance of the homeostatic equilibrium and enabling cell and tissue differentiation [28]. Further these molecular features constitute the prerequisite for an efficient embryonic-maternal communication that is pivotal for the establishment and achievement of a successful pregnancy [29]. Annexin A2 (ANXA2), a calcium-dependent phospholipid-binding protein is a component of the extra cellular matrix and like the *ANXA3* transcript, were up-regulated in this study. *ANXA2* is a pleiotropic gene playing a role in regulation of cellular growing and adhesion and in the transduction of cell signals. It is involved in the maintenance of placentation [30] and is important for embryo adhesion to the endometrium [31]. Cell adhesion is required for embryo morphogenesis [28]. ANXA2 and ANXA1 proteins were decreased in caruncles that were adjacent to the same embryos that were retarded in the present study [13].

Developmental processes were the predominantly altered group in retarded embryos and included nervous system differentiation and functioning. In mammals, neurulation is the next developmental step after gastrulation [32]. The altered transcripts cover a wide range of functions ranging from axonal morphogenesis and synaptic conformation and activity (e.g. neurexin1, neuroglin), growth factor (e.g. neurotrophin) and transcription regulators (e.g. homeobox *LHX8*).

Several other components of the homeobox gene class that were down-regulated in retarded embryos are functionally associated with developmental processes. This is perhaps not a surprising finding considering the role of these proteins as regulative molecules in cell, tissue and organ differentiation. One of these genes, *Goosecoid*, is the first gene expressed in the organizer region of all vertebrates [32]. The homeobox gene *POU5F1* (previously known as *OCT4*), which has a pivotal role in the precocious steps of embryogenesis, and is a marker of cell pluripotency [33], was up-regulated in retarded embryos. These genes regulate the expression of a large number of other transcripts involved in stemness and differentiation, so they need a very fine spatiotemporal tuning. Noteworthy, POU5F1, in coordination with other two pivotal factors (SOX2 and NANOG), represses the expression of genes involved in lineage commitment including also GSC.

Cellular metabolism was another group of altered processes that emerged from the *in silico* analysis. A number of altered transcripts are involved in redox reactions. This class of chemical processes is associated with basic cell biochemical pathways and mechanisms such as energetic metabolism and hormone production. Lipid and steroid metabolism is linked to biological oxidation and hormone secretion and function [34]. The lipid metabolism pathways outlined in Figure 4 highlights the close interplay that exists between the different structural and functional pathways that showed altered expression. The altered transcripts (57) cover a wide range of functions including the homeobox *Goosecoid* (*GSC*) described above. Similarly, amino acid metabolism, including tryptophan and alanine, encompasses important cellular biochemical patterns and influences protein synthesis. Amino acid biochemistry, similarly to other processes of cellular metabolism, constitutes a crucial aspect of in vivo and in vitro embryo development [35].

Complement cascade and coagulation in placenta tissues are known to be involved in pregnancy failure. In humans and mice, altered activity in coagulation and complement cascade, in both maternal and embryonic counterparts, is associated with higher rates of pregnancy failure [36]. Studies in humans (hatching blastocysts, [37]) and mice (foetuses, [38]) showed specific roles for the complement and coagulation cascade in spontaneous loss of pregnancies. In retarded embryos, 15 transcripts were altered, and most (12/15) showed decreased expression. Two members of the SERPIN family (*SERPIND1* and *SERPINA5*) were down regulated and one (*SERPINE1*) was up-regulated in retarded embryos. In the previous proteome study, SERPINE1 was up-regulated in the chorionamnion of buffalo embryos retarded on Day 25 and SERPINA3 (a1-antichymotrypsin) was up-regulated in caruncles adjacent to these embryos [13].

The extensive dysregulation observed in retarded embryos compared with normal embryos suggested that important regulative patterns were altered in retarded embryos. A group of dysregulated transcripts, involved in several processes, code for transcription factors including homeobox (e.g. goosecoid, down regulated), helix loop helix proteins, (e.g. *BHLHE41* (previously known as *BHLHB3*, up regulated), and zinc finger proteins (e.g. *FEZF1,* down regulated). Other down-regulated genes (e.g Neuronatin) are known to be imprinted in several mammalian species [39]. Few non-coding RNAs (ncRNA, e.g. *MIR2315*) were dysregulated; however, it is likely that there was an underestimation of this component because of the lack of full annotation of the transcripts on the bovine array and the heterologous hybridization utilized. Epigenetic mechanisms have been widely established as a pivotal level of transcriptome regulation [40], [41]. Also, there is substantial evidence for a role of genomic imprinting and ncRNA in embryonic differentiation and development [42], [43].

Notwithstanding the limitations of a heterologous system, it does provide an important initial understanding of the transcriptome for species that lack an appropriately annotated genome [23] and the results offer a first insight at large scale level of the molecular events. The heterologous platform chosen for this experiment is enriched for transcripts involved in the bovine reproductive tissues. In our studies, buffaloes that had retarded embryos on Day 25 did not show an increase in circulating concentrations of P4 from Day 10 to Day 20 after mating [3], [13]. Progesterone influences the structure and function of the uterine endometrium, which impacts on the developing embryo [44]–[46]. Accordingly, a lack of progesterone can be presumed to be the underlying cause of retarded growth in buffalo embryos and the differences in transcriptome (present study) and proteome [13] profiles between retarded and normal buffalo embryos. Further evidence of the relationship between P4 and late embryonic mortality in buffaloes is that mortality can be reduced by treatment with GnRH agonist, hCG or P4 on Day 25 after mating [47].

In summary, the present study has demonstrated notable differences in the transcriptome of buffalo embryos that had normal and retarded growth on Day 27 after mating. A major class of altered transcripts were glycoproteins variously involved in membrane structure and function including cell adhesion, ion transport, cell-cell junction and communication, signalling, and secretion. The main biological processes associated with altered gene expression were developmental processes including nervous system differentiation, cellular and chemical homeostasis, ion transport, and general metabolism. We may conclude that changes in the transcriptome of retarded embryos impact on the ability of these embryos to attach to the uterine endometrium and establish a pregnancy.
